# Book Reviews

**Published:** 1822-10

**Authors:** 


### CRITICAL ANALYSIS

OF

### ENGLISH AND FOREIGN LITERATURE

RELATIVE TO THE VARIOUS BRANCHES OF

### Medical Science.

Quæ laudanda forent, et quæ culpanda, vicissim

Illa, prius, cretâ; mox hæc, carbone, *notamus*.—PERSIUS.

### DIVISION I.

### ENGLISH.

ART. I.—*A History of a severe Case of Neuralgia, commonly called Tic Douloureux, &c.; with Observations on that Complaint.* By G. D. YEATS, M.D. F.R.S. Fellow of the Royal College of Physicians, &c. &c. &c. Burgess and Hill, 1822.

ART. II.—*Cases of Neuralgia Spasmodica, commonly called Tic Douloureux, successfully treated.* By BENJAMIN HUTCHINSON, Fellow of the Royal College of Surgeons of London, &c. Second Edition. Longman and Co. 1822.

ART. III.—*Observations on the Nervous System.* By JOSEPH SWAN, Member of the Royal College of Surgeons, and Surgeon to the Lincoln County Hospital. Longman and Co. 1822.

[Concluded from page 240.]

WE have now to lay before our readers an account of the second edition of Mr. HUTCHINSON's work on Tic Douloureux, which, from a pamphlet of seventy pages, has swollen into a volume of 180. On opening it, the first thing which caught our attention was the addition of a plate as a frontispiece, the object of which is thus very fully stated :—"This plate is intended to show the distribution of the nerves principally affected by neuralgia faciei spasmodica, as explained in Mr. Hutchinson's cases of Neuralgia Spasmodica, commonly called Tic Douloureux, successfully treated." On comparing the text with that of the former edition, we find a few verbal alterations and some more important additions. The first of these is a description of the branches of the fifth pair of nerves and portia dura, which occupies five or six pages: these were too well known to admit of any thing new in their anatomical history, although a high encomium is paid to Mr. C. BELL for his new views in regard to their physiology. The next considerable addition is an account of Mr. LIZARS' case, published in the lxixth Number of the Edinburgh Medical and Surgical Journal. The disease affected the third branch of the fifth pair; a portion of the nerve was removed, and the relief from pain lasted for twelve months, at the end of which time the nerve was again divided at the same place, and actual cautery applied to the wound; but without any benefit. "The inferior twig of the fascial nerve, which communicates with the mental, was next divided, but with the same want of success." It was then determined to divide the inferior maxillary nerve before it enters the jaw; and this seems to have been partially accomplished, with considerable difficulty, by means of a sharp-pointed curved bistoury. This likewise failed to remove the complaint. Next day the cautery was applied to the site of the tooth which had been extracted, as the pain seemed confined to this spot, and the patient put upon a course of carbonate of iron for *four* days; a period to the shortness of which Mr. Hutchinson justly objects, as altogether insufficient to form any judgment of its effects. On the 4th of March moxa was applied, and next day the patient was as ill as before. Under these circumstances it was judged expedient to complete the division of the inferior maxillary nerve, where it enters the jaw; and this was done, with great suffering to the patient, by scarifying the bone with a round-shaped gum lancet, between the coronoid process and the internal pterygoid muscle: this at length freed the patient from suffering. Mr. Hutchinson gives Mr. Lizars praise for the ingenuity of his operation, but expresses considerable doubt of the relief remaining permanent; fears in which we participate.

In reference to the case of Dr. Corkindale, giving an account of tic douloureux cured with calomel and opium, a note is added, mentioning that Dr. Leslie, of Pontesford in Shropshire, had met with many instances of this disease in India and Persia, in which he had adopted similar remedies, frequently with success; and that the same results have attended his practice in this country. According to Dr. Leslie, "after the mouth became slightly affected, he seldom observed the disease to continue; but in most cases relief was obtained before six doses were taken." According to Mr. Hutchinson, when mercury "has affected the mouth, the sufferings of the patients have been uniformly aggravated." Now we do not wish to enter into the question of mercury being useful or otherwise in this disease, but to inquire whether ptyalism aggravates the symptoms. In answer to this, we find that the carbonate of iron itself has been observed to produce this effect. "I requested permission," says one of Mr. H.'s correspondents, in allusion to a lady, whose case he relates, "to send her the carbonate of iron, which she granted; when I gave one drachm three times a-day, conformably to your direction, and, upon finishing two dozen powders, she expressed very great amendment, but exclaimed that I had been salivating her: indeed the iron, according to her account, had produced considerable ptyalism. * * * I pressed her not to regard the trifling inconvenience of a *sore mouth*, but to persevere in the use of the powders; which she assured me she would gladly do, as her excruciating torments had been very much relieved." Mr. Hutchinson informs us, in a note, that he has *frequently* witnessed this effect of iron; and we regret that he does not give us more information upon the subject,—especially that he does not inform us whether he, too, has found the salivation from iron accompany the relief in tic douloureux, or whether, like sore mouth from mercury, it has aggravated the pain.

The first parts concludes with two letters,—one from Sir A. Carlisle, the other from Dr. Ash; both of which contain some speculations regarding the nature of tic douloureux.

The six first cases in the second part are the same which appeared in the first edition; they are followed by twenty-one additional cases, five of which only seem to have fallen under Mr. Hutchinson's own observation: these are the xth, xith, xivth, xviiith, and xxivth. The first of these forms so good an instance of the disease, that we shall give it at length.

"Mr. Todd, a very respectable farmer and grazier, residing at Faunfield, in the county of Nottingham, in the twenty-sixth year of his age, of sober habits, and of a delicate lax fibre, began to complain of uneasy sensations in the left side of his face, about a year and a half back, and which he at first ascribed to carious teeth. The pain extended to the left ala nasi, the upper lip, the superior bicuspid teeth, the temple, the upper and lower lip. The infraorbitar nerve, with its manifold ramifications, appeared to be the principal seat of the disease, which consisted of paroxysms, in the periods of recurrence, in degree and duration extremely irregular. They would at times excite only moderate complainings, but more generally the most violent screaming and contortions. The pain would sometimes begin below the eye, and descend to the upper and lower lip. The continuance of these fits of pain was very uncertain, frequently remaining five minutes, and sometimes only a few seconds: a perfect freedom from pain and extreme good health were enjoyed in the happy intervals of disease. The scalding, burning, tearing, and boring sensations, allowed my patient the power of describing the kind of torture with which he was afflicted. There did not appear to be any period of the day in which these paroxysms were more violent, or in their recurrence more frequent. His nights were bad, and the intervals of ease were generally spent in unrefreshing slumbers, alarmed by dreams originating in suggestions of approaching attacks. The paroxysms appeared to come on spontaneously, no external cause exciting them, nor the actions of mastication or of speaking producing this effect. The weaker attacks would frequently admit of alleviation from the compression of the infraorbitar branches, about an inch and a half below the eye. The parts affected always retained their natural appearance, notwithstanding the patient himself always complained of a sense of tumefaction. This train of symptoms clearly pointed out the character of the disease; and the outlines of the case were so strongly marked, that the suggestion of any other malady than the one now treated of was wholly precluded. The plan of cure appeared to my mind equally obvious. Under the mistaken idea that the disease originated in my patient's teeth, he suffered the extraction of three molares, with the mortification of not receiving the most distant relief: on the contrary, an increase of all his tortures immediately succeeded; the violence committed in the neighbourhood of the distracted nerves seemed to aggravate their irritability in a tenfold degree, and his miseries appeared to admit of no alleviation. His complaint was now treated as a rheumatic affection, and various external and internal means were tried with the same unavailing effects. At the expiration of a year's suffering, Mr. Todd applied to me, and I immediately commenced that course which former experience had so frequently taught me to be the most successful. In the middle of last May he began to take one drachm of the carbonate of iron three times a-day, and I directed him to make use of an ointment to the affected cheek every night, consisting of the tartrite of antimony, powdered opium, camphor, and mercurial ointment, one drachm of which was used each evening, until a plentiful crop of pustules appeared; its use was then suspended a few days, and renewed when the eruptions were healed. This plan was duly persevered in for three weeks, when my patient had experienced some very trifling degree of alleviation. The medicines now produced uneasy sensations in the bowels, which were removed, and in future prevented, by mixing the powder with the conf. aromatic, instead of honey; and, if diarrhœa at any time accompanied its exhibition, five drops of the tinct. opii, added to each dose, restrained any inordinate action of this nature. During the ensuing six weeks, my patient pretty regularly persevered in the same plan, modifying and altering the treatment according to existing symptoms, occasionally omitting the medicine a day or two, from unforeseen circumstances. He gradually lost his torments, and is now perfectly well, having seen him only a few hours ago."

In the eleventh case, the patient had laboured under tic douloureux for ten years, and had suffered the extraction of *nine* of his teeth without any benefit. He took one drachm of carbonate of iron twice a-day for five weeks, and his pains were "something alleviated." The dose was then increased to four scruples three times a-day, in which he persisted for two months: at this period "the violence of the disease began evidently to yield to the influence of the medicine;" and at the end of another month his pains left him. Three months after this case was drawn up, the disease returned from exposure to cold, though with very considerable diminution of its violence. But what appeared not a little extraordinary is, that this patient could not be prevailed upon to resume the use of the iron; "he therefore is at times suffering pain." We cannot regard this case as favourable to the cause of iron: indeed, considering that tic douloureux is a disease liable to come on in fits, with irregular intervals of ease, and observing also the length of time the patient took the iron in very large doses before the pains left him, we are much inclined to doubt if the medicine had any share in effecting his recovery. It is obvious the patient regarded his cure with some scepticism, otherwise he would not have declined returning to the use of the remedy on suffering a relapse.

The fourteenth case is an interesting one, and is intended to illustrate the utility of the iron in neuralgic affections of other parts besides the face. The disease seems to have had its seat in the intercostal nerves. A young lady, between twenty and thirty, was attacked, in October 1818, with excruciating pain in the left side, which gradually increased for several months; she lost her appetite, strength, and rest; the slightest touch, or even being moved in bed, excited pain. Leeches were applied to the side, and subsequently blisters, which seemed to aggravate the complaint; she took aperients, the black drop,* bella-

---

* Since these Reviews were written, Dr. Kerrison has politely sent us a copy of his Thesis, from which we take the liberty of making the following quotation on the use of Narcotics in Tic Douloureux:—

" *Extractum Hyosciami* ad grana decem, aut quindecim, intra horas xxiv, dosibus partitis, dolorem plerumque sedat: os, autem, linguam ac fauces arida atque amara reddit. Constipatio non sequitur.

" *Extractum Stramonii* à grani octavâ parte ad grana tria ter quotidie (anno 1817) sumptum nil effecit; vice alterâ, granum vertiginem induxit, cum faucium siccitate, neque levamen attulit tantis molestiis dignum.

" *Extractum Belladonnæ* à grani ¼ ad grana quatuor, ter quotidie. Pharmacum hoc semper ferè neuralgiam facialem spasmodicam debellat, sensilitatem generalem minuendo; redit, autem, dolor simul ac *systema nervosum* statum recuperaverit pristinum. Dosis semigrani, vel grani unius, vertiginem et faucium ariditatem inducit; grana duo, tria quatuorve, tremores, convulsiones et delirium concitant. Alterius dicto fretus, semel accidit, quod tantum hujus medicamenti mulieri dedi, quantum vires ejus sustinere vix potuit (gr. xiv. intra diem,) omniaque mala jam dicta secuta sunt. Nil boni de hoc experimento periculoso evenit; ægrota, attamen, Cinchonâ, aliisque non nocentibus, valetudinem secundam recuperavit, eandemque conservat.

" *Gutta nigra Lancastrensis.* Medicamentum hoc celebre empiricum creditur esse opium, acido acetico solutum, ita ut guttæ singulæ tres valent guttas tincturæ opii usitatæ. *Minima* viginti unâ vice adhibui, atque, dolore urgente, dosin talem ter intra horas xxiv. jussi, ad cruciatum compescendum: neque defuit subsidium speratum. Hujus medicamenti atque opii effectus iidem sunt; quâ de causâ opium semper præponendum est; arcana enim omnia abjudicanda sunt, nisi aliquid utilitatis præstent, medicamento usitato atque cognito haud assequendum.

" *Liquor Opii sedativus.* Hujus inventor non adhuc modum in lucem edidit, quo formatur; nos autem certiores fecit, ut *morphium* purissimum contineat. Nil de usu ejus sequebatur quod non tincturâ opii, pari dosi, meliùs potitum est: liquor enim ventriculo ægrè ferebatur.

" *Opium purificatum.* Non desunt argumenta, cur hoc medicamentum aliis sedativis longè anteponendum est; post multas observationes, tamen, mihi satis cognitum est, quod Neuralgia facialis spasmodica hoc medicamento curari non possit. Alvus adstrictior opio redditur.

" *Camphora* per horas xxiv. semel præscripsi, ita ut ʒss. in misturæ amygdalæ

donna, fetid gums, castor, bismuth, cinchona, and sarsaparilla, all without avail. She now consulted Mr. Hutchinson, who regarded the disease as tic douloureux of the intercostal and cutaneous nerves, and prescribed carbonate of iron; which she continued, he informs us, with some few modifications, until the complaint entirely left her. It appears from the young lady's letter of September, 1821, that she commenced the use of the iron on the 4th of November, 1820, and in six weeks experienced "a trifling amendment" in her side, which induced her to persevere; and in February she was perfectly free from pain, which she had not been, "even for a minute," for two years and a half. She took one drachm of the medicine three times a-day for six months, and then gradually diminished it.

The next case which we shall notice is the eighteenth, which we do not regard as a genuine case of tic douloureux. A gentleman, aged forty-one, whose digestive organs were all along "very much out of order," and who for three months had taken purgative medicines and mercury "to a great extent," became affected with head-ach, entirely confined to the forehead, chiefly seated above the right eye, and accompanied with throbbing in the temples. The pain was sometimes entirely confined to a tooth in the upper jaw, and extended to the nose; it continued sometimes the whole day, at others only a few minutes; but the painful sensation was "*seldom very acute.*" He had "dull oppressive feelings" about the eyes, and was often oppressed with "languor and excessive nervousness." For these complaints, in addition to the remedies already mentioned, he had used arsenic and cinchona, electricity and galvanism, warm and cold baths, general bleeding, cupping, leeches, and, lastly, shaving and blistering the head. He was recommended, by Mr. Hutchinson, to take the usual dose of iron three times a-day, with a tonic mixture containing cinchona, and a pill each night, consisting of Extract. Stramonii, gr. ss; Zinci Sulphat. gr. ij.; Pulv. Rhei, gr. iij.; the bowels being kept open by means of the Decoct. Aloes comp. Mercurials, purgatives, and all medicines likely to debilitate the nervous system, were forbidden, and he was put upon nourishing diet, with a little

---

ʒ xii. probè soluta, dosibus sex partitis sumpta fuerat: Dolore et spasmis uti priùs persistentibus.

"*Fumum Tabaci* per tubum ab ægroto exhaustum, qui fumum haurire non solebat, nauseam et languorem induxit, neuralgiamque facialem minuit, dum fumi effectus narcoticus maneret; exempli gratiâ. Quamdiu vis nervea, hâc de causâ, debilis esset, tamdiù sensilitas nervi dolentis minùs molesta inveniebatur; simul ac, verò, languor abivisset, dolor pristinus reversus est.

"*Acidum Hydro-cyanicum*, secundum Pharmacopœiæ Parisiensis formulam distillatum, per dies plurimos a quodam ægroto sumptum fuerat. Ægrotans, licet effectus ejus omnimodis dignoscere potuit, (si extitissent,) mihi dixit, id nullum præbuisse auxilium."

wine. The bark was omitted at the end of a fortnight, but the other parts of the plan continued for two months, at the end of which time the patient was nearly well, and continued so for six or eight weeks, when he had a recurrence of the symptoms, "but with very diminished energies." The case is a very good one, if it had not been given as an example of tic douloureux: it illustrates well the advantages to be gained by tonics, alteratives, and gentle laxatives, in a patient whose system was reduced and digestion ruined by the continued use, "to a great extent, of purgative medicines and mercury." The patient states that, for three months, he had generally three evacuations in the day, and voided immense quantities of "dark, fetid" mucus, which excited much astonishment; for he tells us that his motions were "composed entirely of this morbid mucus, and notwithstanding the long continuance of the purgatives, *never having omitted them for a day.*" We are much inclined to think that the omission of the purgatives, or at least taking them in a more limited and rational manner, was one of the principal means in effecting his cure.

The last case of Mr. Hutchinson's (Case xxiv.) is one of a very slight affection of the nerves of the face, which was speedily cured by large doses of carbonate of iron.

The remaining sixteen cases were communicated to Mr. H. by practitioners in different parts of the kingdom: many of them are satisfactory, some are otherwise, but the general evidence is very decidedly and strongly in favour of the opinions set forth in this little work. Among other testimonies in its favour is that of Sir A. Cooper, whose letter, being very laconic, we shall insert.

"Dear Sir,—In the few cases in which I have had an opportunity of trying the carbonate of iron, as a remedy for tic douloureux, I have reason to believe that it has a very benign influence in that disorder; and I beg to state, that I was induced to prescribe it entirely from the public recommendation of it which I derived from you.

"I am yours, very truly,

"Astley Cooper."

Upon the whole, we think much credit is due to Mr. Hutchinson for the zeal and industry he has manifested in this investigation; and have no hesitation in regarding the body of evidence as greater in favour of iron than of any other remedy hitherto proposed for the cure of tic douloureux. But, when we observe (p. 15,) that, since the publication of the first edition of this pamphlet, in 1820, Mr. Hutchinson states that more than two hundred cases of this disease have fallen under his own observation, we cannot but wonder at the selection he has made for publication; nor, indeed, can we avoid suspecting that he gives to the term Neuralgia Spasmodica a more general acceptation than we have been accustomed to do in speaking of tic doulouréux. However this may be, Mr. Hutchinson cannot but derive satisfaction from the many and strong expressions of gratitude he has received from those who have been freed from suffering by his advice. Indeed, it is justly observed by the learned and benevolent PALEY, that "remissions of pain call forth, from those who experience them, stronger expressions of satisfaction and of gratitude towards both the author and the instruments of their relief, than are excited by advantages of any other kind." This very circumstance, however, necessarily induces the cool observer to receive these testimonies with some degree of limitation; and we must confess, for our own parts, we should have risen from the perusal with impressions more decidedly favourable, had some of the complimentary parts of the patients' letters been omitted. The remedy, however, is not brought forward as one of specific operation or invariable success.

"Had I (says Mr. H.) advanced the bold and untenable assertion, that the carbonate of iron acted as a never-failing specific in this disease,—that it never disappointed us in curing the most obstinate case of tic douloureux,—or that the malady of thirty years' continuance was equally amenable to its influence as that of only three weeks' or three months' duration, I might possibly have merited this very slight reproof. A professional experience of nearly thirty years, however, has taught me not to place implicit confidence in the curative and specific agency of any remedy at present known. We are not in these days to be taught, that the bark will not always subdue the paroxysms of intermittent fever, and that even mercury has been known to fall short of the subjugation of syphilis. The carbonate of iron has, in a few instances under my own cognizance, been inadequate to the difficult task of curing tic douloureux; an acknowledgment of which is sufficiently manifest in two or three of the preceding cases, as well as in a few others which I have not published. I can, however, assert that, in those very few instances, it has done more towards alleviation than any other known remedy. The carbonate of iron has very frequently been given in very improper cases, and in the most injudicious manner, without any due consideration of peculiarities of constitution, or of the proximate cause of the disease; circumstances essentially requisite to be attended to in the administration of a powerfully stimulant remedy. In the very few unsuccessful cases under my own management, I can almost uniformly attribute the want of success rather to the absence of a due perseverance in the use of the remedy, than to any failure in its power. It ought also to be perfectly understood, that collateral means of relief have uniformly been used in conjunction with the iron, and that the treatment is by no means confined to one remedy. Where symptoms of active inflammation have existed, the means usually employed to subdue inflammation have been had recourse to, and the iron either suspended or never used. I have of late been consulted by many persons, under the injudicious use of iron, when high inflammatory action has existed in the nerves particularly affected, and when the neighbouring parts and general constitution have sympathized with the augmented arterial impulse. To my intelligent readers it is needless for me to explain the results of a practice founded on a complete ignorance of all medical principles. Mr. Abernethy very justly remarks, that 'nerves strikingly resemble arteries in their modes of communication: sometimes they conjoin even by considerable branches, such as must be manifest in common dissections; but they communicate in surprising numbers by their minute ramifications.' This circumstance is not perhaps so familiarly known to professional men, since it cannot be perceived unless in the course of a very minute dissection; and to understand how very numerous these communications are, the representations given by the German authors of their very delicate and laborious dissections may be advantageously consulted."

Mr. Swan commences with some observations on the distribution of nerves, with which it is probable, he remarks, that every organized part of the animal body is supplied; and that the number of nervous branches distributed over any part is in proportion to the degree of sensibility required. First come the organs of sense, over which the nerves ramify in great abundance; and last in order are the ligaments and bones, which are but very scantily supplied. The difficulties which occur in attempting to trace the ultimate termination of vessels which are pervious to fluids, and consequently capable of being injected, are so great as to render our information with regard to the sanguiferous and absorbent systems in this respect unsatisfactory. These difficulties, however, become still more perplexing with regard to the nervous filaments, which, neither being capable of distention nor distinguished by colour, soon become irrecoverably lost in the surrounding textures. To this general ignorance of the termination of nerves, one remarkable exception is presented in the optic nerve, the termination of which in the membranous expansion of the retina probably suggested to Mr. Swan the idea of other nerves ending in a similar manner. He is of opinion that the minute branches of nerves may be traced further by careful dissection than has generally been supposed, and has himself frequently succeeded in following a nerve till it has been lost in a fine membrane. This is said to be particularly remarkable in the face of the horse, and a plate is given to illustrate this piece of comparative anatomy. But it is not merely of the termination of nerves that we are ignorant, —there are certain parts of the body which we rather infer from analogy to possess them, than prove their existence by demonstration; at least, to do so often requires laborious and expert dissection. Mr. Swan has twice succeeded in tracing nerves into bone: in the first instance, the saphænus nerve, which was enlarged from irritation, sent a branch of considerable size into a diseased portion of the tibia; in the second, a branch was given off from the posterior tibial nerve, and distributed upon the fibula. Other instances are adduced in which our author has traced nerves to the periosteum, tendons, ligaments, fasciæ, peritoneum, pleura, and pericardium.

The second chapter is occupied in the relation of a case, which is said to have been disease of the par vagum. A gentleman, aged sixty-two, had been affected with frequent fits of gout, from the early age of seventeen, for which he had been in the habit of using the Eau medicinale, and subsequently Dr. Wilson's medicine, from both of which he procured great relief. The continued use of these remedies seems, however, to have proved injurious to the stomach, the functions of which had become impaired. In November 1820, he began to suffer from difficulty of breathing; and, in the summer of 1821, had a violent attack of this kind, supposed to have originated in exposure to cold: these symptoms were successively removed, either by the supervention of a fit of the gout, or by the vinous infusion of colchicum taken for its cure. The difficulty of breathing, however, returned, which though it again yielded to the colchicum, yet this soon lost its effect, and he suffered more or less from difficult respiration till the period of his death. His stomach remained "craving and insensible;" his body, during many months, became more and more emaciated; his pulse was generally natural, but very strong. A small quantity of blood was taken from the arm ten days before his death, which proved to be very much cupped, and had a strong buffy coat: this depletion produced some temporary relief, but was followed by great faintness next day. At this time he became the subject of experiments suggested by the doctrines which associate nervous influence with galvanism. The result we shall give in Mr. Swan's own words:

"As nothing seemed to relieve him, and as his state appeared to me to be much approaching that of an animal whose eighth pair of nerves had been divided, he was galvanized. He thought the two first trials gave him some relief. A few nights before he died he was taken with difficulty of breathing to such a degree, and seemed so exhausted, that some wine was given him, and galvanism was again tried. After the first ten minutes, the noise in his breathing left him, and he kept breathing more and more easily, so that, when the galvanism had been used for half an hour, he laid down and slept better, for several hours, than he had done for some time before. The galvanism was repeated the next day, and he thought himself relieved by it: but this relief was of short duration, for his breathing soon became as bad as ever, and he died a few days after, on the 22d of September."

The diseased appearances are thus described;

"On opening the abdomen, every thing appeared sound. The outside of the stomach was covered with an unusual quantity of veins, but the inside of this viscus had nothing particular in its appearance.

"On opening the chest, there was much fat. The heart was enlarged and fat, but otherwise every thing about it had a healthy appearance. In the pulmonary artery, one corpus sesamoideum was larger than the rest; and, in the aorta, one corpus sesamoideum was not situated at the edge of the valve, but about its middle.

"Each side of the chest contained about two pints of a dark-coloured fluid. The lungs were not collapsed, but appeared otherwise healthy.

"On tracing the par vagum from the middle of the neck, each nerve was flabby, and much smaller than natural, and felt like nerves removed from a putrid body after having been soaked in water. The branches distributed to the lungs appeared as is usual; as did the continuations of the nerves, nearly as far as the termination of the œsophagus, when they were found redder and thicker than usual, and had not a healthy appearance. The left nerve was smaller than the right.

"In order to be better satisfied with regard to the diminution of the size of the par vagum stated in the preceding case, I was led to compare the appearance of it, as there related, with that in other cases; and, in dissecting two subjects destroyed by consumption, the left lungs were diseased in a much greater degree than the right; both trunks of the par vagum were smaller than they should be, and especially when compared with those of a subject destroyed by empyema where the lungs were sound; and the left trunk was smaller than the right. In both of these subjects, as well as in another, very considerable disease existed in the intestines. In one, I examined the alimentary canal from one end to the other; and through its whole length, beyond the stomach, very little space was left where ulcerations of the mucous membrane did not exist, most of them varying in size from a pea to half-a-crown.

"Respecting such a dreadful disease as consumption, I think any facts are of importance; and it might be well worth while to step out of the beaten track, and inquire what is the deficiency in the body which exists as the predisposing cause.

"I have stated the above facts, respecting the par vagum, as they presented themselves to me. Whether they have been only accidental occurrences, or the usual concomitants of similar diseases, must be left for future observation to determine."

Now, how far the par vagum may become diseased, either as cause or effect, in hydrothorax, must be left for future observation to determine; but we cannot agree with Mr. Swan in his reasoning upon this case, when he says, "As I have before observed, the symptoms never were those of hydrothorax:" whereas, it appears to us that there were several circumstances strongly indicative of that disease. "The difficulty of breathing continued with different degrees of violence till the time of his death." "He had a cough, which was occasionally troublesome." "Within the last three weeks, he had been obliged to rise in the night, and sit up a great part of it." There was some swelling of the legs, (attributed by Mr. Swan to the position of the limbs;) and, though last not least, there were found two pints of fluid in either side of the chest. Besides, when we consider that dyspeptic symptoms, such as are described in this instance, attend almost every case of gout, we cannot perceive why we should seek for any other explanation of them. Mr. Swan argues (p. 15,) that the craving for food, and want of a sensation of fulness, showed the nerves "had lost their sensitive qualities;" forgetting that (p. 12,) his patient had taken an emetic, "in consequence of which his stomach had ejected a quantity of food;" an infallible proof, in our opinion, that the nerves continued to act. Again, referring to the account of the dissection, we confess ourselves extremely sceptical as to the accuracy of the assertion that the par vagum "was flabby;" the expression, when applied to nerves, being at best but indefinite. But when we are informed, further, as something worthy to be noticed, that one nerve was smaller than the other, we are tempted to be angry with Mr. S. for trifling with his readers; this appearance, so far from arguing disease, being one of the most common possible: indeed, we are convinced the description of the best anatomists will bear us out in asserting, that the par vagum varies so much as scarcely to be found identically similar in any two individuals.

The chapter entitled "on Ulceration of the Nerves," is chiefly occupied by the relation of two cases, in neither of which is it very clear that ulceration of the nerves had occurred. They were fungous ulcers on the legs, with which the nerves going to the neighbouring parts were connected. The first example occurred in an unmarried woman, aged forty-three, of strumous habit, and, all remedial applications having proved unavailing, the limb was amputated. On dissecting it, the external popliteal nerve was observed to be much larger in the lower part of the ham than it was higher up; the cutaneous branch became quite blended with the posterior part of the fungus, after passing which it regained its natural appearance. The anterior tibial nerve, as it approached the diseased part, became covered with a firm and very vascular membrane, which followed it through most of the fungus, after which the appearance of the nerve became more healthy. The fibular nerve exhibited appearances similar to those just described:—"at the beginning of the fungus it gave off to its base many branches, which were very much *enlarged* in consequence of irritation."

It will be observed that here, as in several other places, Mr. Swan talks of enlargement of the nerves from irritation; but it is to be remembered that size is a relative term, and we are therefore at liberty to adopt our author's opinion or not, as we find most consonant with probability. We doubt if the enlargement to which he alludes was connected with the fungous ulceration, because careful examination has been made, within our own personal knowledge, of nerves leading to cancerous or other painful ulceration, without any enlargement being found; and because the most violent irritation of nerves, as in tic douloureux, does not lead to their enlargement; neither does the most protracted instances of convulsive diseases. If irritation of nerves produced enlargement, their quiescence might be expected to produce an opposite effect; but this is contrary to fact, for the nerves of paralytic and the corresponding sound limbs have been carefully measured, without any difference being detected; a circumstance which Dr. Powel stated, in his Lectures at the College of Physicians, that he had verified by many examinations at St. Bartholomew's Hospital.

Mr. Swan is of opinion that the excruciating pain frequently attending fungous sores, is caused by the inflammatory action extending to the nerves in the vicinity, and on this principle suggests the expediency of cutting out a portion of the nerve which supplies the fungus, in the same way as the nerves have frequently been divided for other causes. A case having presented itself of extensive and very painful ulceration connected with the external popliteal nerve, a portion of it was cut out in the following manner:—An incision, two inches in length, was made along the inner edge of the outer hamstring: this divided the skin and fat, and brought to view a thin tendinous fascia, on dividing which the nerve was immediately exposed. First a probe, and afterwards a probe-pointed bistoury, was passed under it, and the nerve divided as near as possible to the superior part of the wound; about an inch was removed, and the edges of the wound brought together with adhesive plaster and a bandage. After the nerve was divided, the pain in the ulcer entirely ceased, and the patient had no feeling on the top of the foot. "The wound made by the removal of the nerve hardly caused any inconvenience. After the operation, he never had any of the spasms in the limb, or any of the violent pains which followed the course of the sciatic nerve, and caused so much suffering. His state was rendered much more comfortable by the operation, but he still at times suffered pain from the connexion of the saphænus nerve with the ulcer." Unfortunately, however, the bone was extensively diseased, and amputation became necessary. On dissecting the limb, the saphænus and sciatic nerves were found *enlarged*; and the fibular nerve was *enlarged* and much thickened at the place where it had been divided. A new branch went from the point of division to the anterior tibial nerve. "The junction (says Mr. Swan,) of this branch with the anterior tibial nerve was at the inside of the nerve; and, from the manner in which it is situated, I think it not improbable that some other branches, forming a medium of communication between the divided portions of nerve, might have been destroyed in the dissection. New branches went from the same portion of the divided nerve to the fibular nerve and to the surrounding parts." These branches, however, were flatter than nerves of the same size usually are; and, although Mr. Swan regarded them as new *nerves*, yet he acknowledged his inability to ascertain to what extent they possessed the power of conveying nervous influence. Although there can be no doubt that nerves, under favourable circumstances, are capable of reproduction, yet it does not follow that the restoration of continuity between the divided portions necessarily arises from structure similar to other portions of the nerve. In examinations of this kind, it would be well to keep in view the tests proposed by Meyer, in his "Essai sur la Regeneration des Nerfs;" these are, 1st, that nitric acid destroys the neurilema, but does not act on the medullary structure of nerves; and 2d, that the functions of a nerve, having been destroyed by its division, can only be renewed in consequence of the continuity being restored by a similar production. This last, it must however be confessed, is not satisfactory.

After relating a painful paralytic affection of the hand, in which, after amputation, the median nerve was much enlarged, and several of the digital nerves, towards their terminations, had a gangliform enlargement, Mr. Swan proceeds to speak of Tic Douloureux. Under this name he seems to class all painful affections of nerves. Of the two cases he relates, one was irritation of the sciatic nerve, from pressure caused by an aneurism, the operation for which cured both complaints; the other seems to have been neuralgia of the second cervical nerve, and removed by bark, blue-pill, and tincture of muriate of iron. His ideas of the proximate cause of the disease are spoken of elsewhere.

Chapter vi. treats of "Dizziness:" it is very short, and the subject discussed in rather a vague and unsatisfactory manner. Our author is of opinion that this symptom is, in many instances, continued merely from habit; and that, although the patient may have been relieved at first by bleeding, yet that further loss of blood, so far from relieving, will aggravate the complaint. Others, he remarks, instead of dizziness along with it, have unpleasant feelings about the head, with debility, palpitations, restlessness, and "mental irritation amounting almost to insanity;" all which he has known cured by means of bark, wine, and generous diet. Now, with regard to the latter series of symptoms, we apprehend that very few practitioners would think of having recourse to blood-letting, any more than Mr. Swan: with regard to the former, it is well known that vertigo frequently arises from indigestion, and is to be most easily alleviated by remedies tending to improve the state of the stomach and general health. But where the patient has, "in the first instance, been relieved by bleeding," and "should the complaint soon return," we cannot but surmise that doses of bark and wine, with generous diet, appears to us a doubtful method of cure; and it is in not pointing out sufficiently the symptoms which justify the exhibition of the tonic medicines he has recommended, that our author has been deficient. A young practitioner, were he to be guided by what Mr. Swan says on this subject, would be very apt, in an attack of "dizziness," to follow up the bleeding (if he *did* bleed,) with bark and wine; and, if this dizziness arose from determination of blood to the head, (certainly the most frequent cause in those cases which fall under our treatment,) the mistake would most likely prove fatal.

The three remaining chapters treat of "Diseased Appearances in the Spinal Canal," "Injuries of the Medulla Spinalis," and "Paraplegia." The only part of those which our limits will permit us to notice is a very interesting case, which, but for its length, we would give without abridgment.

Colonel Sibthorp, aged forty-three years, was overturned in his carriage, February 23d, 1821. He was visited about an hour after, when he was cold, faint, and complaining of great pain between the scapulæ, but of none in the head; he could not move the left leg at all, and the left arm but very little; he had a tingling sensation in both arms; the paralyzed leg became affected with cramp to such a degree as to render it necessary to hold it. He had no difficulty in passing his urine, but it did not flow very freely. When pressure was made on the left side of the spinous process of the first dorsal vertebra, it excited much pain; not so if pressure was made on the right side. Twelve ounces of blood were taken from the arm, and an opiate administered. Next day, it was deemed necessary to repeat the bleeding to the same extent as before. Towards evening he was more comfortable; the spasms of the left leg were less frequent, and the tingling in the arms less distressing. From this time to the 4th of March, he had leeches occasionally applied to the spine; took moderate doses of calomel and other purgatives; under which treatment he gradually improved: he had not, however, sensation in the little finger, or inner side of the ring-finger, in the right hand, so perfect as in the other parts. April 1st, was able to walk a little. In May, he complained of numbness in the right hand; when the spine was again examined, but no irregularity was perceptible: nor, indeed, was any crepitus, or any other symptom, observed at the time the accident occurred, which could lead to the supposition that any of the vertebræ were fractured; unless the pain on pressure, above mentioned, can be regarded as such. It is worthy of remark, that the leg which had been completely paralyzed improved much faster than the arm, analogous to what we observe in hemiplegia from effusion in the head.

"From the nature of the accident, the patient's amendment was greater in the time than might have been expected. I met him very frequently walking in the streets at Lincoln at the latter end of the summer, and, though his left arm continued in a considerable degree paralytic, I felt confident that time would gradually restore him the use of it; and in this opinion Dr. Cookson and my brother concurred with me.

"About the middle of September he went to London, and was advised, by the medical man he there consulted, to submit to a peculiar mode of treatment, in consequence of being told by him that several of the vertebræ were dislocated, or compressed inwards, and that the paralysis of the arm depended on this dislocation; that, by the peculiar process recommended, and the use of lubricating liniments, the bones might be restored to their proper places; and that then the paralysis would cease.

"As part of this mode of treatment, the patient was pulled and pressed for about an hour, almost daily, for several weeks; and such was the violence of the pressure, that on one occasion, in particular, something cracked, and it was believed at the time that a rib was broken, for immediate pain was produced, which continued several days. My medical readers will form their own judgment as to the effects likely to be produced by such a process as this. For myself, I have no hesitation in thus publicly avowing, that I all along expressed to those who asked me, that, in my opinion, it was utterly impossible thus to have restored the parts to their proper position, supposing any dislocation had really taken place, which, however, I was confident was not the fact; and further than this, that, in an operation attended with so much violence, there was great risk of injury to the general system, in a case where, from the nature of the accident, the parts were rendered more susceptible of morbid changes. But, whatever the opinions of others might be, in the judgment of the medical gentleman himself the process adopted was completely successful: all the bones were said to be put into their proper places, and the patient almost, if not entirely, cured; debility alone remaining, which, as the cause was stated to be removed, it was expected would soon cease also.

"He reached home on the 5th of December, and I visited him on the 7th. He at that time complained of vomiting, head-ach, impaired vision, and constipation of the bowels. He said his left arm was nearly in the same state as when he went to London, and, if it was improved at all, it was not in a greater degree than he thought it would have been in the same time provided nothing had been done.

"He seemed desirous of trusting himself to the *vis medicatrix naturæ*, as, in consequence of what had recently occurred, he had, with very good reason, a great aversion to the interference of art. He continued nearly in the same state until the 25th of January; the alvine evacuations were then black, and, common aperient medicines being of little use to him, he took submuriate of mercury until his mouth became sore, when the colour of his evacuations changed, but his other symptoms did not abate. His sight had become gradually worse; he had continual sensations in his eyes, as if flashes of light were always before them, but the pupils contracted and dilated properly. He said he felt a general weakness. He made water with difficulty, and experienced the same want of power in expelling the fæces. It was feared that disease was going on in the membranes of the medulla spinalis and brain; and leeches, blisters, and setons were recommended to him, but their application was not consented to."

February 2d, he complained of pain in the back of the neck, extending over the head, accompanied with difficulty of swallowing; which symptoms, however, again diminished, and he seemed improving till the 26th, when he was taken in the night with head-ach, vomiting, and difficulty of breathing; his lower extremities began to swell, and his left arm and leg became affected with involuntary movements. These symptoms continued to get worse till the 9th of March, when he died, having previously become nearly blind, and been much convulsed.

On examination after death, no appearance of dislocation or of fracture was to be seen: "the spinal canal was perfectly natural in every part, and no pressure could have been on the medulla in any part of it." The chief appearances of diseased structure were in the tunica arachnoides: in the brain, "the whole tunica arachnoides had an opaque appearance;" in the medulla spinalis, it was "thickened and very opaque." Near the termination of the medulla, there were contained, under the arachnoid coat, several small eminences, about the size of pin's heads; three inches and a half at the lower end appeared firmer than natural and thickened, containing, very near the termination, a solid tumor, semi-transparent, and about the size of a very small pea. About four ounces of fluid were found on each side of the chest, and a small quantity in the pericardium. The abdominal viscera, generally speaking, were sound; and, on removing them, the bodies of the vertebræ were found to be exactly in their places.

The case is followed by some general observations, and by remarks upon the minute anatomy of the medulla spinalis and brain.

"To observe the gradual diminution of the energy of the nervous system, and at last its almost total extinction, and to find after death no materially diseased part, except the tunica arachnoides, and that only changed from its healthy appearance by being a little thickened and opaque, would naturally lead to one or other of these two suppositions: either that this membrane is in itself a very important agent in the nervous system; or that it is an index to show the state of the parts with which it is connected, and which, in point of fact, are labouring under the same disease, though so constructed as to escape observation. Now, with regard to the former of these suppositions, if it were true that the tunica arachnoides was spread only loosely over the medulla, as on a superficial examination it appears to be; and yet the change of appearance in it, which I have described, had naturally produced the violent symptoms in it stated in the preceding case; undoubtedly it must be looked upon as of such importance, from its extreme sensibility of morbid changes, as to render it impossible for any one to have the nervous system perfect when this membrane was in any way diseased. But, in direct opposition to this, in the instance of James Cawthorn, where we might reasonably suppose this must have been the case from the number of cartilaginous deposits in the tunica arachnoides, we know that no impaired functions were the consequence. I am, therefore, strongly inclined to favour the latter of the two suppositions I have stated: and, indeed, I am much disposed to believe that the tunica arachnoides is not merely a loose covering of the medulla, but that it is reflected over both the inside of the dura mater and the exterior surface of the pia mater. It appears to invest the pia mater closely, and to be reflected from its posterior surface to form the loose covering. The points of reflection are remarkable, and appear like adhesions between the tunica arachnoides and pia mater; and, even though the membranes should be merely united at these points, I conceive that the connexion between them is such, that one could not suffer a considerable diseased action for any length of time without affecting the other. In the examination of Benjamin West, I observed a spot of cartilaginous matter on the dura mater, exactly like those on the loose portion of the tunica arachnoides; and, from its being on the inner surface, I was led to conclude that it was situated on the tunica arachnoides lining the dura mater. It is contrary to the order of the animal economy for two parts or membranes, which are merely in contact with each other, to be of dissimilar structure and functions: indeed, the two different surfaces would be continually irritated by the secretion of each other, and perpetual inflammation would be the consequence, until an adhesion had been produced between them. To account, therefore, for the symptoms in Colonel Sibthorp's case, I should say, that the diseased appearances of the tunica arachnoides were merely indicatory of a similar disease in the more vital parts of the medulla, with which it is connected. I think it most probable that, when the tunica arachnoides becomes changed from inflammation, whether chronic as in this case, or acute as in the case of Cornelius Bishop's son, that the pia mater partakes more or less of the same action. The pia mater is so closely connected with the medulla, and contains so many blood-vessels, as to be very unfavourable for marking those delicate changes observable in the tunica arachnoides; but which, even in this latter membrane, would be frequently overlooked, were it not for its natural thinness and transparency. The medulla is composed of a very beautiful and delicate cellular texture, within the cells of which the medullary matter is contained. The cells are attached to, and indeed are, I apprehend, a continuation of the pia mater, or at least have their origin from it. From hence, should the same change of structure take place in the pia mater as in the tunica arachnoides, we may readily conceive how it might be communicated to the cellular structure containing the medulla, and thus impair its natural powers; and that this might go on in the same degree as in the loose tunica arachnoides, and yet be so little marked in its appearance in the pia mater and the delicate cellular structure, as to escape the utmost penetration of the anatomist.

"The intimate structure of the medulla is of very difficult discovery. I have in vain attempted to find it out mechanically. I could observe all the parts anatomists have described, but of the medulla itself I could, by dissection, discover no more than that it was a pulpy mass. Finding thus that I could not succeed by the aid of instruments, I had recourse to chemical agents. I divided a portion of the medulla spinalis of the ox longitudinally, and immersed it in the liquor potassæ of the London Pharmacopœia. After some days, the medullary matter appeared to be dissolved, when, by adding water to it repeatedly, it was washed away and suspended in the water, so as to form a fluid like milk. What remained had the appearance of membrane. I suspended this in water, and found it to be that beautiful and cellular structure which I have just mentioned. I afterwards put other portions of the spinal marrow into liquor potassæ for twenty-four and forty-eight hours; I then washed them with water, so as to dislodge part of the medulla, and immediately after put them into rectified spirit of wine, which hardened the medullary matter left in the membranes; I then suspended them in water, and found the same beautiful and cellular structure empty, and continued from the portions containing the medullary matter. After I had done this in the ox, I could make out the same structure in the human subject. The cineritious substance is far more delicate than the medullary. The same structure exists in the brain, but it is very difficult to make it out so distinctly; and, contrary to the opinion of some anatomists, I find the structure of the medulla of the spine is of a much firmer nature than that of the brain."

At the end of the volume are eight plates. The first of these represents the membranous appearance of the nerves on the face of the horse; the second, which is very badly coloured, a portion of the gastrocnemius muscle, with the same membraneous or plexiform appearance; third, the tibia and fibula receiving filaments of nerves; fourth, nerves going to the tendons and to the pericardium of a calf; fifth, distribution of nerves on the upper surface of the foot; sixth, the anterior tibial nerve sending numerous filaments to the base of a fungus; seventh, different views and sections of the fungus; eighth, showing the state of a nerve some time after its division; various twigs going from the divided extremity to the surrounding parts; ninth, a portion of the median nerve, showing its connexion with the sheaths of the tendons. We have to object to these plates, that they seem rather to be plans or diagrams than natural representations of the parts.

Mr. Swan informs us, in the preface, that it is his "utmost wish to be useful to the profession in which he is engaged." Now, we give him full credit for his sincerity, and think, besides, that he has both zeal and talents to realize his wishes; but we do not think he is taking the most judicious method of accomplishing his end. In 1820, Mr. S. published "a Dissertation on the Treatment of Morbid Local Affections of Nerves," which was reviewed in this Journal in the Number for April 1821. On that occasion, our predecessor demonstrated the lack of originality in the work, by comparing it with that of Mr. Pring, published five years previously; and, on perusing the present pamphlet, (for, exclusively of the plates, it occupies only ninety-three pages,) we are struck with the silence observed with regard to what others have written on the subject of which he treats. Possibly this may not be from ignorance or neglect of their opinions; but, on the other hand, if so, "it hath not appeared." We think the cases he has published in what he himself calls their "present imperfect state," might have formed an important addition to the second edition of the work above mentioned; but that they will add nothing favourable to his reputation ushered into the world in the form of a separate volume, which is left to find the level of its own merits.

---

### DIVISION II.

### FOREIGN.

Art. III.—*Traité des Maladies des Artisans, et de celles qui resultent des divers Professions, d'après* Ramazzini. Par Ph. Patissier, Docteur en Medicine, &c. 8vo. pp. 420. J. B. Baillière, Paris.

This work, which is professedly written upon the model of Ramazzini's "Diatriba de Artificum Morbis," but greatly extended, and adapted to the improvements that have taken place in the various branches of medical science, particularly chemistry, consists of an introduction of some length, and a classification of the subject matter under three principal heads or divisions; the first of these classes being subdivided into five orders, the second admitting of no subdivision, and the third class comprising four orders. This arrangement, which is in a great measure the same as that employed by Fourcroy, is not essentially different from that of Buchan; and, in those points in which it differs, it may be said that it also degenerates; for, according to the plan that our author has adopted, repetitions without end are produced, and the bulk of the volume is unnecessarily increased. The practical utility of the work is also much diminished by his mode of describing the diseases, in too many instances, without noticing the method of cure; and he has also employed by far too much of his space in detailing the processes of many of the manufactures already well known and detailed in most elementary chemical works. The quantity of original matter contained in this volume bears but a small proportion to that which the author has extracted from contemporary writers; but he deserves the praise of having acknowledged handsomely all he has borrowed, and has indeed been studiously careful to mark all those passages taken from Ramazzini especially. We must, however, protest against his frequent quotations from Buchan, whose work he appears to consider as of very high authority.

Having made these few preliminary remarks, we shall now proceed to give a concise, but faithful, account of the contents of this book.

The Introduction comprises fifty-nine pages, the object of which is to present us with a sketch of what has been written upon this subject, both before and after the time of Ramazzini. The following extract from a paper by M. Cadet Gassicourt is not uninteresting, as a specimen of opinions relative to the moral tendency of certain employments, which, though in many respects coinciding with our experience on this side of the Channel, may be suspected of being a little too refined in some particulars.

"Thus," says he, "common opinion attributes to butchers a cruel and sanguinary character; but an appeal to the registers of the police, the tribunals, and the prisons, will show that fewer acts of violence are committed among these men than among bakers, who are not believed to possess dispositions so cruel. By similar researches, I discovered that a seditious and turbulent spirit belonged principally to masons, but especially to printers; that the lowest debauchery was practised by shoemakers; that pedrasty was the crime of boys at lemonade shops, of barbers, and waiters; that braziers were distinguished by avarice; that prudence, good order, and docility, were met with commonly among gold-beaters, basket-makers, and goldsmiths. The morality of artisans is commonly in proportion to the degree of instruction they receive, to the quantum of their wages, and to the healthy nature of their occupation." (p. xxxi.)

The insalubrity of certain trades, and the actual disease, as well as great nuisance, which they occasion in the midst of large cities, has long been a subject to which the government of France has paid particular attention; and, by a decree of the year 1810, the most offensive and pernicious were prohibited from being established within a certain distance of any large town. This remark naturally leads to the consideration of the influence that epidemic diseases exert upon particular employments: thus, in the plague at Marseilles in 1720, all the bakers perished; and M. Desgenettes, in his Medical History of the Army of the East, observes that bakers, cooks, blacksmiths, and all those workmen who were exposed to great and sudden changes of temperature, contracted the plague with more facility than others.

The fourth section is devoted to a consideration of the comparative mortality of different callings. One general remark will serve to illustrate this branch of inquiry,—that, wherever the occupation is most dirty, and the wages are the lowest, the proportion of death will be the greatest; sedentary occupations, and working in close and crowded situations, of course, conjoined, materially influencing this proportion. M. Villerme remarks, that those men who are accustomed to work in the open air bear sickness with impatience, and more readily sink under disease than those whose occupations are usually sedentary.

A very curious table follows, containing a list of all the artisans admitted into the different hospitals in Paris during the year 1807, and giving the proportion of deaths. It is much too long for insertion, but it illustrates the general observations before noticed, and confirms the statements made by Dr. Beddoes as to the general good health of butchers. This table would have been more perfect had there been any means of knowing the actual numbers of the different trades, so as to have formed a judgment of the proportion of sick from each.

We shall conclude our extracts from the introduction, by informing such of our readers as may be ignorant of the fact, that, within these few years, societies, closely allied in their plans to our benefit societies, have been formed among the working classes in Paris: they amount now to about 120 in number, and consist of upwards of 40,000 members.

We now come to the consideration of the first class of diseases, namely, those caused by the fumes of metallic substances, which leads to a discussion of the means by which the inspiration of the noxious vapours or particles may be prevented. To all these schemes the same objection applies, that they guard the mouth, but leave the nostrils unprotected; added to which, the inconvenience of wearing masks, however composed, or of breathing through tubes, to men employed in laborious occupations, will always prevent, in our apprehension, their general adoption; to say nothing of the prejudices usually entertained by people in these situations of life. None of these objections, however, apply to the furnace invented by M. Arcet, of the royal mint at Paris, the sole effect of which is to increase the draught of the chimney, and, by the assistance of a ventilator in the window of the apartment, to impel the noxious vapours up the chimney. M. Arcet described this invention in a little work, entitled the Art of Gilding Bronze, published at Paris in 1818. It is found so efficacious, that no gilder is now permitted to change his workshop without constructing one of these furnaces in his new residence. This invention is due to a maker of gilt bronzes, of the name of Ravrio, who left a bequest of 3000 francs to the person who should discover the best method of preserving the workmen from the pernicious effects of mercurial vapours; and the reward was adjudged to Mr. Arcet.

The long section devoted to the diseases and accidents of miners, presents us with no new facts that need detain us for a moment. The safety-lamp of Sir H. Davy is mentioned with deserved praise, and its employment in mines strongly urged.

Among the diseases to which gilders on metal are subject, the mercurial trembling, as it is termed, is by far the most common and fatal. Its attack is usually by slow degrees: a slight trembling of the arms is first perceived, and, if the workman continues to pursue his occupation, temporary loss of recollection, watchfulness, and delirium; the agitation of the limbs at length becomes convulsive. Nevertheless, the appetite is not much impaired; the bowels continue to perform their functions regularly; and they generally recover if they cease their occupation. The curative means seem to consist, indeed, of little more than change of employment, and the exhibition of sarsaparilla. It is, however, asserted, that the invention of Mr. Arcet has rendered this affection extremely rare.

We have a long account of the painter's cholic in the subsequent section, with a description of the mode of cure as practised at La Charité. We find nothing here, however, to extract: the treatment of this affection is well understood. It appears that the proportion of deaths from this disease at La Charité amounts to 1 in 34, nearly.

It is stated, as a distinction between this disease and the cholic to which the workers of copper are subject, that, in the latter, the retraction of the abdomen and the obstinate constipation are not so frequent: in short, the attack partakes more of the character of inflammation, and fever is present. (p. 79.)

Respecting the diseases produced by the neighbourhood of salt-works, some diversity of opinion prevails. The town of Cervia, on the coast of the Adriatic, furnishes salt to nearly the whole of Italy: it is considered so unhealthy, that the popes granted it as an asylum to those who wished to escape from their creditors. Other situations where salt is manufactured, however, contradict this opinion, and would lead one to suspect other causes for the insalubrity of that place. In the vicinity of Venice, for example, much salt is made, and no inconvenience is observed. The writer of this article passed the greater part of a year in the town of Augusta, in Sicily, in the immediate vicinity of which there are extensive salt-works, yet no ill consequences appeared to result to the inhabitants; for the malaria fever, as it is there called, is common to the whole coast, towards the latter end of summer.

The second order of diseases, comprising all those produced by the inspiration of animal effluvia or particles, occupies a space of eighty-five pages; but, in fact, all the accidents and complaints included in this long list of trades and professions may be reduced to a very few heads: for example, the accidents to which nightmen, well-diggers, and those who cleanse common sewers, are liable, may all be reduced to two kinds; the one arising principally from the evolution of ammoniacal gases, among the former class of workmen, and known by the name of *mitte*; the latter the effect of azotic, or sulphurated hydrogen, gases, and called, in the language of the Parisian workmen, *plomb*. The former of these affections is only a temporary evil, causing sharp pains in the eyes, which appear red. There is also an unpleasant sensation of stoppage in the nostrils, and a pain which strikes from the bottom of the orbit to the forehead. A total or partial blindness, which lasts two or three days, is often conjoined with these symptoms. Confinement to bed for a few days in a dark room, and cold lotions applied to the forehead and eyes, soon remedy this inconvenience. The attacks of asphyxia, to which all the various classes of these workmen are subject, are of a nature infinitely more serious: they admit of division into two kinds,—the one produced by the sulphurated hydrogen and sulphuiated hydrogen ammoniacal gases, and which is distinguished by convulsive motions: the other, the true asphyxia from the absolute want of respirable air, the effect of azote.

The symptoms distinguishing the effects of the former of these gases, are violent pains in the stomach and in the joints; a sensation of choking; involuntary cries; delirium; sardonic laughter; general convulsions, and death. M. Hallé mentions the case of one unfortunate man, who suffered from both kinds of attack on the same night. These gases are seldom met with in emptying the liquid matter; but the mere movement of the solid contents often disengages it. Where it is found, bodies in a state of combustion burn more brightly.

The effects of the nitrogen gas are the reverse of this: it extinguishes flame, and death ensues quietly, and without convulsions.

The greatest danger often occurs when the matter has been entirely removed, and when water escapes into the pit. M. Dupuytren relates an accident of this kind, where, some days after the emptying a privy, a workman descended to examine the walls, a little water having got in; twelve or fifteen hours after, the same man again descended, and fell immediately dead; those who went to his assistance were seized with asphyxia, one after the other. Three cases, taken from the records of the Hotel Dieu, are here inserted, in corroboration of these facts, and their treatment detailed: and we must here remark, that the inhaling chlorine, the bleeding, and the affusion of cold water afterwards, are, in our opinion, but doubtful remedies; and we should be rather inclined to say, instead of two of the men being cured by them, that they got well in spite of them. The third man died. The dissection was only remarkable on account of the very rapid decomposition of the body which had been effected forty hours after death, (in the month of September.) All the viscera were gorged with blood, and the stench was so horrible, that the assistants, even to the keeper of the amphitheatre and his wife, were more or less affected with cholic or vomiting, which lasted in some degree during the whole day. (p. 114—121.)

The remedy which the workmen commonly employ in these cases appear to us much more rational, and is pretty uniformly successful: they give stimulants immediately, and then endeavour to excite vomiting as quickly as possible. It is probable that the *immediate* effusion of cold water on the chest and body, by exciting the action of the abdominal muscles and diaphragm, and thereby emptying the chest, might be attended with success: it is very simple, and can almost always be immediately procured. Among the precautions against these accidents we find nothing novel.

The diseases of makers of catgut, tanners, curriers, and flayers of horses, and other offensive trades, may be dismissed with a few words of comment. Those who work in the open air do not appear to suffer any other inconvenience in consequence of their peculiar employment, farther than hard labour, confinement to particular postures, or exposure to damp or moisture, may be supposed to give rise to. The men employed in dissecting-rooms are asserted, by our author, to die generally of aneurism, or ruptures of the heart: their gross habit of intoxication, and the heavy weight of the dead bodies they are accustomed to lift and carry, are supposed to predispose them to these affections. Two or three cases of this kind are detailed. (p. 156.)

M. Husson observes, that those who are employed in winding off the cocoons of silk-worms die frequently of pulmonary consumption; and that fowls that pick up and eat the dead bodies of the silk worms, lay eggs the odour of which is intolerable.

We know not how the corporation of cooks may relish the advice that is given them, to take purgative medicines frequently, in order to preserve the sensations of the palate "*dans toute sa virginité;*" but we believe few English cooks would consent to acquire celebrity at the expense of their stomach and bowels.

On the subject of punctures to which anatomists and surgeons are occasionally liable, our author quotes the opinion of M. Laennec, that simple ablution with cold water, and suffering a stream of water to fall into the wound, is more efficacious than the destruction of the parts by caustic; a doctrine from which we beg leave to dissent.

We must pass over many of the remaining sections with very slight notice. In mentioning the diseases to which medical men are most liable, the exemption of the majority of them from plague and fever supposed to be contagious, may fairly be ascribed to the short time that the medical man passes with each patient,—to the trifling degree of real compared with the apparent danger, if the breath of the patient and the exhalations from his body be avoided,—and to the little fear usually entertained by practitioners.

Beyond the use of personal cleanliness, frequent changes of linen, good nourishment, and abundant exposure to the open air, there would seem to be no decisive means of avoiding infection. The first of the rules that M. Fodere lays down with great gravity, as proper to be observed by medical men upon such occasions, is to proceed to one's business as if there was no danger; which is evidently impossible to a man whose mind is already alarmed, and a very useless rule where no previous apprehension exists. Our author makes a remark in this place, the truth of which will be acknowledged by many a student:—"La plupart des jeunes médecins croient être atteints des maladies dont ils font une étude particulière; nous en avons connu plusieurs qui s'imaginoient être affectés d'aneurysmes du cœur, ou des gros vaisseux, de la phthisie pulmonaire, et laryngée."

We proceed now to discuss the third order of diseases, caused by vegetable vapours or particles, which will not detain us long, most of the maladies included under this head originating rather from the mechanical irritation of the particles than from their peculiar nature. Not so, however, are the attacks which those employed in the making of wine are liable to; for, independently of the state of half-intoxication which this employment commonly produces, they are too often seized with asphyxia, arising from the evolution of carbonic acid gas. This vapour may be seen hovering over the vats like a dense cloud; and, if a lighted candle be brought near, the flame soon becomes yellow and feeble, and at length is extinguished. A

person exposed to this vapour falls down instantly, and perishes if not promptly assisted: his first sensation is a numbness of the legs and arms, a feeling of constriction about the chest and throat, a confusion of the senses, and a suspension of respiration first, and then of the circulation. This accident is so common, that, our author says, there is no farmer in the wine-countries that has not a recollection of such an event among his neighbours or relatives. The best method of recovery in these cases is to bring the patient into the open air, to throw fresh water, or vinegar and water, into the face, to use frictions to the chest, and to inflate the lungs if respiration is not speedily re-established. (p. 224.) The first part of this treatment is that which has long been practised at the Grotto del Cani near Naples; the dogs which are the subject of the experiment being always thrown into the adjoining lake. The best mode of preventing this accident is to avoid placing too many vats in the same cellar; not to have them placed too high from the floor, or too near each other. The workman should not stoop at his work, and he should never visit the cellar alone. It must be recollected that the maker of cyder is subject to the same accident.

We may omit noticing the remaining orders of this class, as relating to subjects familiar to the profession. This brings us to the second grand division of the work, which treats of those diseases brought on by exposure to damp. Our notice of this portion of the volume will be short, there being fortunately no subdivisions. Under the head of diseases of seamen, however, M. Patissier contrives to fill some pages with the instructions for preserving their health published by Captain Cook: but what he can mean by the following foot-note, it is beyond our ability even to guess:

"Le scorbut de mer est si terrible, qu'on a cru devoir en faire une espèce distincte, et le separer de celui de terre; mais tous les bons medecins conviennent aujourd'hui que ces deux espèces ne different point l'une de l'autre, et qu'on les combat avec succès par les mêmes remèdes."

If the author here means to assert that the different species of purpura, which certainly were in many instances formerly confounded with scurvy, and have even occasionally of late years been loosely called "land scurvy," are to be all treated alike with a vegetable diet and acid drinks, he certainly is on this point much mistaken, and has not exerted his usual industry in collecting and recording those facts and observations that illustrate this now well-understood branch of cutaneous diseases.

The last class of this numerous catalogue of complaints now offers itself to our notice. It is entitled diseases arising from defect or excess of exercise; and hence it will be evident that much must have been anticipated in the former part of the work,—an ample illustration of the objection we made *in limine* to the arrangement altogether. We must agree, however, with our author in condemning a prejudice of the "great vulgar," that those who are under the necessity of working hard for their living are equally long-lived with those more fortunately circumstanced. Labourers are, in general, very old men at sixty; soldiers and sailors, at a much earlier period of life. A list of those diseases to which the labourer is subject comprehends, indeed, some of the most terrible maladies to which humanity is exposed: hernia, aneurism, ruptures of blood-vessels, and all kinds of inflammatory affections are included in this list. M. Corvisart asserts that couriers, and those who travel great distances on horseback, are extremely liable to diseases of the heart. He quotes the example of a man who had travelled on horseback upwards of one thousand miles without repose; besides this, he performed a journey from London to Paris, and in crossing from Dover to Calais (it is not stated whether he suffered from sea-sickness or not,) he felt, for the first time, a difficulty of breathing, and spat blood. In spite of these symptoms he continued his route; he became much worse by the time he reached Paris. He was bled five times within three days, without relief. The following days, (how many is not said,) he became agitated in a violent manner; he appeared suffocated, and died. On opening the left ventricle, one of the fleshy pillars that support the mitral valves was found ruptured at its base; there was also an appearance of suppuration upon that spot. (p. 291-2.) It is easy to conceive that the over-exercise of any particular organ may induce disease; and, therefore, that those who are called upon to exert their voice should be subject to inflammatory affections of the larynx, spitting of blood, and consumption, is not at all remarkable. The same may be said of those professions in which the fixed and constant use of the organ of vision is called for.

It would appear that those workmen who are obliged to exercise their callings in an erect posture, are more particularly subject to varices and ulcers on the lower extremities, to pains in the loins, and weakness of the stomach.

We must now take our leave of M. Patissier, rather abruptly perhaps; but we have already devoted so much space to him that we have none left to take a ceremonious leave. We have endeavoured to give a faithful account of what is interesting in his work; we have used no more freedom with him than our duty to our readers rendered it incumbent upon us to do; and we leave him with the assurance that we shall meet him again with pleasure.

[We have been induced to deviate a little from our usual plan, by inserting the following remarks under the head of Critical Analysis, in consequence of the novelty, as well as the great importance, of these experiments.]

ART. V.—*An Account of some Experiments on the Nerves;* by M. MAJENDIE. *With some Observations,* by JOHN SHAW, Esq. Lecturer on Anatomy in Great Windmill-street.

In the last Number of the Journal de Physiologie, several very curious experiments are related by M. MAJENDIE, which are not only important in themselves, but are interesting, as they corroborate some experiments which had been previously made in this country; but of the performance of which M. Magendie does not appear to have been aware. Perhaps, the detail may be acceptable to some of your readers.

*Experiments upon the Functions of the Roots of the Spinal Nerves.*

"For a long time I have been desirous of discovering what effects would be produced upon an animal by dividing the posterior roots of the spinal nerves. I have made frequent attempts to cut those twigs, but they all proved unsuccessful, in consequence of the great difficulty which is met with in opening the vertebral canal, without at the same time so injuring the spinal marrow, as either to destroy or to be productive of great torture to the animal. In the course of last month, however, a litter of eight puppies, about six weeks old, having been brought into my rooms, I thought they afforded a good opportunity for renewing my attempts to open the vertebral canal. With one stroke of the knife, I succeeded in exposing the posterior half of the spinal marrow, enveloped with its membranes; and I had only to divide the dura mater which surrounds it, to lay the whole organ completely bare. By this method of proceeding, I had under my eye the posterior roots of the lumbar and sacral nerves; and, by raising these alternately, with a pair of fine scissars I could easily divide them upon one side, without at the same time affecting the spinal marrow.

"I had not entertained the slightest idea of what would be the probable result of this experiment; and, after the wound had been closed by the application of a suture through the skin, I observed the effects produced on the animal. I was fully persuaded, at first, that the division of the nerves had completely paralyzed the member which they supplied; for, in fact, it showed no sensibility, either when pricked or violently pinched, and was to all appearance totally motionless; but soon, to my surprise, it began to move very distinctly, notwithstanding the sensibility of that part was quite extinguished.

"In a second and third experiment, I was presented with exactly similar results; and it then occurred to me as more than probable that the posterior roots of the vertebral nerves were possessed of functions distinct from the anterior, and that they were the nerves which were destined for sensibility.

"It was a natural suggestion, in order to make good this conclusion, to cut the anterior roots only, and leave the posterior ones undivided; but this I discovered was more easily imagined than performed: for at first sight it appeared to me an impossibility to expose the anterior part without injuring also the posterior roots. This, however, did not deter me from inventing plans to overcome the difficulty, and at length, after two days' consideration, I determined to pass a kind of cataract knife in a direction anterior to the posterior roots. From its blade being very narrow, I expected that, by directing the sharp edge along the posterior face of the bodies of the vertebræ, I could by this means divide the roots. This plan, however, I had to resign, on account of the large veins which are contained in this side of the canal, and which were opened by every cut that was made in a forward direction.

"During these attempts, however, I discovered that, by tearing off the dura mater from the spinal marrow, I could display the anterior roots, united in bundles, just where they begin to pierce that membrane. This was all I could desire, and in a very few seconds I succeeded in dividing all the pairs of nerves which I wanted. In this, as in the preceding experiments, I divided those on one side only, in order that I might have a better opportunity of observing the effects by the contrast.

"It is easily conceived how curious I was to discover the effects of this last experiment; they were distinct, and left no room for doubt: the limb was altogether deprived of the power of motion, and totally powerless, yet its sensibility was evidently preserved. In short, to make the matter perfect, I divided both the anterior and the posterior roots in the same animal; the consequence was a total loss both of sensation and the power of motion.

"These experiments I have repeated and varied upon different animals, and the results, both with regard to the anterior and posterior roots, were confirmed in a manner the most satisfactory. These researches are to be continued, and in the next Number I shall offer a more detailed account of them. For the present let a positive assertion be sufficient, that the anterior and posterior roots arising from the spinal marrow possess different functions; and that to the posterior more particularly belongs sensibility, while the anterior seems to be more especially connected with the power of motion."

The importance of the facts discovered by these experiments must be evident to every one; and it must be gratifying to the true friends of science in this country to find that M. Majendie, whose sole object in these pursuits appears to be the promotion of physiology, has, by his experiments, come to the same conclusions as those which had been previously deduced by Mr. CHARLES BELL, from observations made on the anatomy of the brain and spinal marrow. The truth of these deductions were also by him put to the test of experiments; the results of which, however, though they corresponded with those of M. Majendie, were not so conclusive. As I have entered into some speculations on the same subject, I shall, perhaps, be excused in bringing before your readers the following extract from the last volume of the Medico-Chirurgical Transactions:

"I shall, however, take the liberty of trespassing still more upon the time of the Society, by making a few remarks upon a very curious question, which has particularly excited the attention of physicians in all ages since the time of Galen:—Why sensation should remain entire in a limb, when all voluntary power over the action of its muscles is lost; or why muscular power should remain when feeling is gone?

"The attention of Galen was particularly directed to this question, in consequence of his having been called upon, by some of his contemporaries, to account for the manner in which he had cured a partial paralysis of the finger by applications made to the spine.

"In answer, Galen told them that two sets of nerves went to every part; one to endow the skin with sensibility, the other to give the muscles the power of voluntary action. This opinion was probably founded on a mere theory; but the facts lately discovered, and the observations which have been noted in attending to the phenomena of disease, though they do not afford absolute proofs of the correctness of Galen's supposition, still they go far to establish the fact, that every part of the body which is endowed with two or more powers is provided with a distinct nerve for each function.

"The form of the nerves which at the same time endow the skin with sensibility and the muscles with the power of voluntary motion, is such that they appear to be single cords; but, if we examine the origin of any of those nerves, we shall find that it is composed of two packets of fibres, which arise from distinct parts of the spinal marrow. These origins are soon enveloped in the same sheath, so as to appear to a superficial observer to form a single nerve.

"It is not too much to suppose that either of these origins may be affected while the other remains entire. To prove this by ocular demonstration will perhaps be impossible, and therefore the question will probably remain undecided. But we have already seen examples of the consequence of injury to a nerve that has a single root, viz. the portia dura; for, if we cut it, there will be only one set of actions paralyzed; while, by dividing a nerve which has a double origin, viz. the fifth, we shall destroy two powers, viz. voluntary motion and sensibility. We know, also, that, when we cut through the trunk of a nerve going to the hand, we destroy both sensibility and the power of motion.

"In reference to this subject, I shall state the result of certain experiments which were made about thirteen years ago, by Mr. Charles Bell. The two sets of filaments by which each spinal nerve is connected to the spinal marrow were exposed: on irritating one set, convulsion of the muscles upon which the nerve was distributed ensued; but, when the other was excited, no perceptible effect was produced. These experiments we have often repeated, and always with the same results; but, from the violence necessarily used in making them, it has been difficult to ascertain which of the filaments bestows sensibility on the part. It was easily shown that, if only the posterior set was destroyed, the voluntary power over the muscles continued unimpaired; but the pain necessarily attendant upon the performance of the experiment prevented us from judging of the degree of sensibility remaining in the part.

"It was, I believe, the result of these experiments which induced Mr. Bell to give an opinion nearly similar to that of Galen, in a short essay on the Anatomy of the Brain, which was printed and distributed among his friends in 1809. An opinion somewhat similar has been lately offered by Dr. W. Phillip, in answer to a query of Dr. Cooke's.

"If the view which I have here taken of this question be correct, it may lead to this rule of practice. If only one set of functions of a spinal nerve be deficient, we should apply our remedies to that part of the system from which the nerve arises; but, if both functions are impaired, we must then direct our inquiries to the state of the nerve in the whole course, from its origin to its distribution, as the loss of power is probably owing to some affection of a part of the nerve, after the two sets of filaments by which it arises are united together."

As I have stated, the experiments alluded to above have been often repeated, and always with the same results. I shall here copy verbatim the notes which I drew up on the 21st of March, 1821, as memoranda of the result of some experiments* made with Mr. Bell upon that day, to be inserted in his private journal.

"But this part of the research (viz. certain experiments on the ninth and glosso-pharyngeal nerves,) was hurried in order to examine the spinal marrow. The bones of the spine were exposed at the lower part of the neck, by cutting through the muscles, &c. as quickly as possible; about two inches of the canal were exposed by sawing through the crura of the spinous processes. The proper sheath of the spinal marrow was not opened; but, the whole being pulled a little to one side, the nerves were seen going off. On irritating the posterior origins, no convulsion was produced; neither was there any on pinching the ganglion: but, on pinching the posterior roots, or on pinching the two sets of origins at the same moment, a convulsion took place in the corresponding muscles. This was repeated on several of the nerves, and in all with the same results. Several of the pupils, who were assisting, were satisfied of there being a marked difference between the two sets of fibres: indeed, it appeared almost quite satisfactory that there was no convulsion produced by irritating the posterior roots or the ganglion.

Mr. Hawkins, who was my principal assistant, makes the following observations, (and which I at the time copied into my notes:)—"The spinal marrow was exposed immediately after death, while some of the muscles had still a slight convulsive

* These were experiments Mr. Bell was unwilling to repeat often on living animals, after having seen the effect once or twice on them; he contented himself with ascertaining the effect after the animal had been stunned.

motion. Upon irritating the posterior root of the spinal nerves in three or four places, no effect was produced upon the neighbouring muscles; but, when the anterior root singly, or the whole spinal nerve, was pinched by the forceps, or pricked by the scissars, an evident motion was produced on the muscles, not only perceptible to the eye, but, when the third or fourth dorsal nerve was touched, the whole scapula moved in the hand of the assistant. This motion was not communicated to the muscles when the ganglion which is formed on the posterior root within the sheath was touched; neither did it follow an injury of the posterior column of the spinal marrow. Whether an injury of the anterior column of the spinal marrow would produce convulsion, was not ascertained, as it was not cut upon till the irritability of the muscles appeared to be exhausted. The motion given to the muscles was not the slight tremulous motion arising from the natural irritability still remaining in them, but it was convulsive and spasmodic, and followed each successive prick of the scissars."

The importance of these experiments, especially since their results have been corroborated in so extraordinary manner by M. Majendie, will, I expect, be soon made apparent to every one; for the accumulation of facts will probably embolden Mr. Bell, in a short time, to give an account of his investigations into the anatomy of the spinal marrow, and the views he has deduced from them, which will lead to an entirely new doctrine of the anatomy of the brain and nerves.*

When upon this subject, I cannot avoid taking the opportunity of making a few comments upon the result of some other experiments which are connected with the inquiry.

M. Majendie has taken much interest in the investigation of the functions of the nervous system, and, in several of the late Numbers of his Journal, he has given an account of the experiments which were instituted by Mr. Bell, to ascertain the correctness of certain views deduced from the discoveries which he had made in the comparative anatomy of the nerves. M. Majendie has also repeated several of the experiments, and with the same results, excepting his having found that the act of mastication was less impaired on cutting the infraorbital nerve than what is stated in the relation of the first experiments performed here. In a note, in the Number for October 1821, to an account of the result of certain experiments which I had

---

* In justice to Mr. Bell, I should state that this has been a subject of constant contemplation with him for the last fifteen years. He has thought it necessary to rouse attention to the subject of the nerves, by first offering his papers on the Respiratory Nerves, and on those of the Orbit and the Throat, before that on the General System. It has been in vain that we have urged the danger of anticipation.

detailed to him, he says, "Le resultat que nous avons obtenu s'accorde parfaitement avec celui que nous venons de rapporter a l'exception toutefois de l'influence de la section du sousorbitaire sur la mastication, influence qui n'a pas été evidente pour moi."

In my communication to the Journal of Science, No. xxiv. I made the following observation on M. Majendie's objection:—"The idea that we had, in our experiments in England, found the power of mastication destroyed, must have arisen from the difficulty I had of expressing myself correctly in French, when discussing the results of the experiments with M. Majendie. There is *one act* of mastication destroyed by cutting the infra-orbital nerve; but, to destroy the power altogether, it would be necessary to cut the trunks of the fifth nerve on each side." Since that paper was published, I have made several experiments, which, at the same time that they prove the correctness of M. Majendie's observations, also establish, in the most satisfactory manner, one of the most important discoveries of Mr. Bell,—viz. that the fifth nerve and the spinal nerves are of the same class, and that they in every respect correspond with each other. At present I shall not enter fully into this question, but only state the following circumstances, which will probably be acknowledged to be correct:

1. That the head and face, having many parts in every respect similar to the neck, trunk, and limbs, must have corresponding nerves.

2. That the manner in which the spinal nerves and the fifth arise by double origins, is very similar.

3. That the two origins of the fifth are united by a ganglion exactly of the same shape and character as those which unite the two origins of the spinal nerves.

4. That the manner in which the branches of the fifth are distributed, and those of the spinal nerves, is the same.

And, lastly, with reference to the anatomy, we find that the same kind of connexion exists between the fifth and the sympathetic, as between the latter and the spinal nerves. In their morbid affections, the similarity also holds good: thus, in the common cases of hemiplegia, the spinal nerves and the branches of the fifth are similarly affected. In this disease, the voluntary power over the limbs and the sensibility of the side affected are generally destroyed; but in some cases the voluntary power is lost, and the sensibility continues unimpaired, or *vice versa*. This variety also occurs on the face; for the jaw will drop, and there will be all the marks of paralysis, but the sensibility of the skin and the sense of taste will continue entire.

In experiments on the nerves of the spine and on the fifth, we meet with the same results. If, as in the operation which is now frequently performed on the nerves of the horse's foot, we cut a spinal nerve after the branches are given off to the muscles moving the part, we shall destroy only the sensibility of that part; but, if we cut the nerve nearer to the brain, we shall not only destroy the sensibility, but all the power of motion. The same happens in experiments on the fifth; for, if we cut a branch which is principally distributed to the skin of the lips, we shall destroy the sensibility of the part, but impair the power of mastication only in a slight degree, (and this, as I have already remarked, M. Majendie has observed and commented on;) but if we divide the nerve further back, then we shall not only destroy the sensibility of the skin, as in the first experiment, but also cut off the power by which the jaws are moved. I cut a branch of the fifth upon the face: the sensibility of the corresponding side of the lip was destroyed, but little paralysis ensued. I cut the nerve nearer the brain, and at a point previous to its having given off the branches to the muscles; then the jaw fell, and the muscles of that side were powerless. I varied the experiment, by irritating the nerve where it lies in the spheno-palatine fissure, immediately after an animal was killed: the jaws then came together with much force, indeed, so as to nip my assistant's finger severely. This last experiment may be compared with the very common one of galvanizing the nerves which pass from the spinal marrow to supply the muscles of the extremities.

When the inquiries with which we are now occupied are farther advanced, I shall enter more minutely into this question: at present I shall only offer you some general observations upon the difference between the original, or spinal nerves, and those of the superadded class.

To distinguish the superadded nerves from those of the spine and the fifth, we may first look to the facts afforded by comparative anatomy,—viz. that the first class corresponds to the number and complication of the superadded organs. If we, then, examine the anatomy of the nerves forming this class, we shall find that, instead of their rising by double origins,—i. e. by distinct roots from the two great spinal columns,—they each arise by a single series of fibrils, from a distinct portion of the spinal marrow. There is no ganglion on the roots; at least, there is no ganglion at all similar to those which are found on the fifth or on the spinal nerves: (it is ridiculous to compare the swelling on the par vagum, after it has emerged from the skull, with the ganglion which connects the roots of the spinal nerves.) The manner in which the superadded nerves are distributed is also different from that of the spinal nerves.

If, in the living animal, we expose a spinal nerve, and one of the superadded class on the face or side of the neck, and irritate them, we shall have no difficulty in determining that the spinal nerve is the most acutely sensible to pain: indeed, in the greater number of the experiments which I have made, the degree of sensibility of the superadded nerves seems to be so slight, when compared with that of the spinal nerves, as to make it a question whether they be at all sensible.* If, after an animal is dead, we stimulate a nerve of each class, either with the common pincers or with the galvanic forceps, we shall be convinced that the nerve of the superadded class continues the longest to influence the muscles. But the greatest and most marked difference between the two classes is discovered by observing their natural functions, and the phenomena of disease. Let us take any one of the superadded nerves, the portio dura, for example, which goes to the eyelids, nose, and mouth: by a voluntary effort, we can close the eyelids, or we can frown; but is either winking or that action of the eyelids and brows which is so marked during mental emotions, or the closing of the eye during sleep, governed by any power of volition? We can move our lips at will; but is smiling or laughing, or the action of the lips during anger, under our controul? or can we whistle or blow, unless the actions of the lips and cheeks are in unison with the respiratory apparatus of the throat and chest? We can turn up the tip of the nose, or we can pull it down; but can we command the muscles of the nostrils during violent respiration? What are we to think of the blindness of those who cannot discover the strong actions produced through this nerve in the muscles of the nostrils and lips, during that state of apoplexy where all voluntary power is lost? All these complicated functions will be destroyed by cutting the portio dura; whereas, if we only cut the fifth, which gives branches to all the parts that the portio dura does, these various faculties will continue unimpaired, while the sensibility of the parts, and a set of voluntary actions quite distinct from those already enumerated, will be destroyed.

If we examine the actions of those parts of the throat and neck which are supplied with branches from the par vagum, we shall discover that they are endowed with the same variety of voluntary and involuntary powers;† and, what is still more

* Some difficulty arises here from our mistaking compounded nerves for simple ones. On one occasion I was nearly drawn into a mistake by not observing that a small filament of the fifth nerve entered into the portio dura on the face. There may be branches of the nerves of simple sensation combined with those of the eighth and ninth. See Note to my first communication to the Journal of Science.

† The almost complete destruction of the power over the lips as manducatory organs in those animals which graze, by cutting the portio dura on either side, makes the analogy between the par vagum and the portio dura more striking. The par vagum not only regulates the actions of the larynx and pharynx in breathing, but also in swallowing; so it would appear that the portio dura, in some extraordinary, we shall find that the actions performed through the medium of the spinal accessary are of the same character. Thus, by a voluntary act, we can raise the sternum in a long inspiration; but, if we put our fingers on the sterno-cleido mastoideus, and snuff quickly up, we shall find that we cannot prevent the muscle from acting; and this involuntary action of the sterno-cleido is still more evident in sighing, sneezing, or coughing.

The phenomena presented when the superadded nerves are affected by disease, are in every respect different from those displayed when the fifth or spinal nerves are in a similar state. Upon this subject I shall not enter, but refer to the several papers which have been already published in the Philosophical Journals.

It would, perhaps, be well to make a short summing-up of the discoveries referred to in this sketch, but I will defer this, in the hope that you will, in a succeeding Number, permit me to offer you a short view of the whole question. Indeed, such a statement is now in some degree necessary, as those who are unacquainted with the immense variety of subjects involved in the inquiry may, perhaps, be swayed by some insidious attacks which have been lately made upon it, and particularly by one who, it will scarcely be believed, was for some years under Mr. Bell's roof.

Though I am personally interested in the question, in consequence of the part I have taken in the performance of the experiments by which the views deduced by Mr. Bell from comparative anatomy were substantiated, still it is with much unwillingness that I take any notice of a late pamphlet: for, when engaged in such a delightful inquiry, it is most unpleasant to be involved in a dispute with one whose object seems rather to be an attempt to detract from the merit of his late master, than a wish to promote the interests of science.

I shall not enter into a refutation of the inferences which the author of the pamphlet has drawn from the experiments, because, to those who are acquainted with what has been done in Windmill-street, during the last three years, in the prosecution of the anatomy of the nervous system, it must at once be evident that the results of the experiments repeated by the author with the hopes of overturning the great discoveries made by his master, instead of answering his intent, afford additional proofs of the correctness of those views, which were originally drawn from anatomy. This consequence the author could not have anticipated; for, if he had, the spirit which seems to have instianimals, not only gives the muscles of the nose and mouth power as respiratory, but also as manducatory organs. This question involves a very curious and interesting subject, to discuss which would far exceed the limits of a communication.

gated him in writing the pamphlet would have prevented him from lending any assistance to the inquiry; though, indeed, the spirit of hostility displayed by him makes that evidence important, which would otherwise be of little value.

To those who may suppose, from the manner the author has announced *his* experiments, and particularly those on the eyelids and tongue, that he has the merit of originality, I shall only make this request, that they will look over my papers which have been for some time published in the Medico-Chirurgical Transactions and the Journal of Science.*

To those who are anatomists, it is needless to point out the incorrectness of the critique upon the origin of the par vagum. It cannot have been written in ignorance of the great difference which there is in the origins of the spinal nerves and the par vagum, as there is at this moment in the museum of Great Windmill-street a preparation made, under Mr. Bell's directions, by the author of the pamphlet, to show the very distinction which he attempts to confound.

To those who are yet unable to form a judgment of the merits of the question, the last paragraph of the pamphlet will be sufficient evidence of the spirit which instigated the author when writing it.

---

MONTHLY

### MEDICAL AND SCIENTIFIC BIBLIOGRAPHY.

* The only *new* experiment, (if so it may be called,) among so many claimed as original, is that of cutting the portio dura on both sides, instead of only on one side, as we had done. The results of this experiment, instead of being contradictory of any circumstance stated to have occurred when only one was cut, confirms in a more curious manner the truth of the view which had been deduced from comparative anatomy, of the use of the portio dura, but which the author, from his ignorance of the subject, does not appear to have been able to comprehend. (See note to page 350.)

